# Pharmaceutical Metabolism in Fish: Using a 3-D Hepatic *In Vitro* Model to Assess Clearance

**DOI:** 10.1371/journal.pone.0168837

**Published:** 2017-01-03

**Authors:** Matthew G. Baron, Kate S. Mintram, Stewart F. Owen, Malcolm J. Hetheridge, A. John Moody, Wendy M. Purcell, Simon K. Jackson, Awadhesh N. Jha

**Affiliations:** 1 School of Biological Science, Plymouth University, Devon, United Kingdom; 2 AstraZeneca, Alderley Park, Macclesfield, Cheshire, United Kingdom; 3 School of Biomedical & Healthcare Science, Plymouth University, Devon, United Kingdom; University of Siena, ITALY

## Abstract

At high internal doses, pharmaceuticals have the potential for inducing biological/pharmacological effects in fish. One particular concern for the environment is their potential to bioaccumulate and reach pharmacological levels; the study of these implications for environmental risk assessment has therefore gained increasing attention. To avoid unnecessary testing on animals, *in vitro* methods for assessment of xenobiotic metabolism could aid in the ecotoxicological evaluation. Here we report the use of a 3-D *in vitro* liver organoid culture system (spheroids) derived from rainbow trout to measure the metabolism of seven pharmaceuticals using a substrate depletion assay. Of the pharmaceuticals tested, propranolol, diclofenac and phenylbutazone were metabolised by trout liver spheroids; atenolol, metoprolol, diazepam and carbamazepine were not. Substrate depletion kinetics data was used to estimate intrinsic hepatic clearance by this spheroid model, which was similar for diclofenac and approximately 5 fold higher for propranolol when compared to trout liver microsomal fraction (S9) data. These results suggest that liver spheroids could be used as a relevant and metabolically competent *in vitro* model with which to measure the biotransformation of pharmaceuticals in fish; and propranolol acts as a reproducible positive control.

## Introduction

The study of pharmaceuticals and personal care products (PPCPs) in the environment has prompted significant attention due to their potential for inducing both short and long-term biological effects in aquatic organisms [1]. As these compounds are designed to act on specific therapeutic targets (e.g. enzymes, transporters and receptors) in humans [2], that are often conserved across vertebrate phyla, it is possible that inducible effects demonstrated in human target systems may also induce similar effects in non-target organisms, such as fish [3, 4]. Additionally, the continued presence of these contaminants in the aquatic environment gives rise to the potential risk of accumulation in fish and other aquatic organisms [5–7], although as yet not clearly established outside the laboratory. Since the adoption of the United Nations Stockholm Convention on persistent organic pollutants (POPs) in 2001, there has been significant activity concerning the assessment of the number of persistent, bioaccumulative and toxic (PBT) substances worldwide [8]. In addition, there have been extensive calls from regulations such as REACH (Registration, Evaluation and Authorisation of Chemicals) to avoid unnecessary testing of these substances on animals in line with commitments to and support of the 3Rs (Replacement, Reduction, Refinement) initiatives. Instead to utilise existing information from standard and non-standard methods, *in vitro* methods, read-across and weight-of-evidence approaches, in an integrated testing strategy for assessing the PBT nature of these substances [9, 10].

Conventional alternative bioaccumulation assessments for fish rely mainly on *in silico* approaches such as Quantitative Structure Activity Relationships (QSAR) that relate molecular properties of a compound to a measure of a particular activity (i.e. acute toxicity) [8]; perhaps due to the cost and labour intensity of performing large-scale *in vivo* bioaccumulation tests [11]. Unfortunately, these computational predictions are not validated for many chemical classes, including pharmaceuticals, and more importantly they do not account for the impact of metabolism in the organism [12]. In fact, these estimates of bioaccumulation can be significantly affected by an organism’s ability to metabolise a chemical [13, 14]. It is assumed that metabolism (biotransformation) of a xenobiotic is likely to result in improved clearance. In recent years, the use of *in vitro* techniques in the bioaccumulation assessment of PPCPs has received more attention, with a particular focus on the measurement of metabolism and the extrapolation of this data to an *in vivo* scale, to aid improved predictions of the bioconcentration potential of these chemicals [15]. In particular, the use of three-dimensional (3-D) hepatic fish cultures or ‘spheroids’ has been proposed as an alternative model with which to assess metabolism, efflux and bioaccumulation potential of PPCPs in aquatic environments due to their *in vivo*-like physiology [10, 16–18].

As *in vitro* studies with fish hepatic models can be used to support screening-level bioaccumulation assessment of contaminants [2, 13, 14, 19], the aims of the present study were as follows: (1) determine the metabolic competency of 3-D liver spheroids prepared from rainbow trout, a recommended regulatory fish species, towards selected environmentally relevant pharmaceuticals; (2) utilise this *in vitro* data to (a) make predictions on pharmaceutical metabolism in fish based on ‘read-across’ to human metabolism data and (b) calculate intrinsic clearance rates for liver spheroids to compare with values obtained from both fish and human *in vitro* studies.

## Materials and Methods

### Ethics statement

This *in vitro* model of fish liver spheroids utilises primary liver cells derived from freshly killed rainbow trout, *Oncorhynchus mykiss* (Walbaum), supplied from the fish husbandry facility of the AstraZeneca Brixham Environmental Laboratory. The fish were held with permission from the UK Home Office within compliance with the AstraZeneca Global Ethics Policy. Prior to cell harvest, the fish were killed humanely under Schedule 1 of the Animals (Scientific Procedures) Act 1986. Since the fish were not exposed to any test compounds, and that individual animals were used to test multiple substances, we believe this work contributes to replacement and reduction of animals compared to standard methods of *in vivo* exposure.

### Pharmaceuticals, chemicals and reagents

Atenolol (purity ≥98%; CAS 29122-68-7), metoprolol succinate (purity ≥98%; CAS 98418-47-4) and propranolol hydrochloride (purity 99%; CAS 318-98-9) were obtained from AstraZeneca (Alderley Park, UK). Diclofenac sodium salt (purity ≥98%; CAS 15307-79-6), phenylbutazone (purity ≥98%; CAS 50-33-9), carbamazepine (purity ≥98%, CAS 298-46-4) and diazepam (purity ≥98%; CAS 439-14-5) were purchased from Sigma-Aldrich (Poole, UK). All chemicals and reagents for tissue culture procedures were obtained from Life Technologies Ltd (Paisley, UK). Pharmaceuticals were prepared fresh on the day of exposure in solvent (DMSO) and diluted in Leibovitz’s L-15 medium (no serum or antibiotic addition) to a concentration of 200 μg L^-1^ (0.2% DMSO). A final well concentration of 100 μg L^-1^ (0.1% DMSO) for each pharmaceutical was used. Pharmaceuticals that carried a salt weight were accounted for when calculating exposure concentrations i.e. final well concentrations refer to parent chemical minus the counter ion.

### 3-D liver spheroid culture

Maintenance of female diploid rainbow trout [*Oncorhynchus mykiss* (Walbaum)] (wet weight: 116.0 ± 21.7 g) and liver dissociation procedures are described previously [10]. Individual livers (wet weight: 1.43 ± 0.34 g) produced a sufficient cellular yield to use single fish replicates, thus each fish was used as an individual experiment. Cell viability after isolation was determined by a trypan blue exclusion test (0.2% final volume; L-15 medium) and cell suspensions with a viability of ≥ 85% were used for spheroid culture. Penicillin-Streptomycin (5,000 U mL^-1^) and amphotericin B (250 μg mL^-1^) were added to the culture medium (1% v:v) in addition to foetal bovine serum (FBS; 10% v:v). Cell suspension aliquots (1 X 10^6^ cells mL^-1^; 3 mL volume) were transferred to wells of Poly(2-hydroxyethyl methacrylate) (pHEMA)-coated six-well micro-plates (Falcon, VWR, UK) and placed on an orbital shaking platform (Innova 2000, Eppendorf, UK), set at a constant rotation speed of 70 RPM. Plates were maintained at 15 ± 1°C in a temperature controlled laboratory. Culture media was replaced every two days until spheroids reached maturity (8 days). A more detailed protocol for cell isolation and spheroid formation is described previously [10].

### Preparation of spheroids for exposures

Spheroids (8 d) were pooled (for each individual fish liver) from 6-well micro plates, into a pHEMA-coated 50 mL centrifuge tube and washed three times with 10 mL L-15 medium (no serum or anti-biotic mixture; spheroids were allowed to sediment without centrifugation prior to each washing step). As spheroids are too large to be counted on a haemocytometer, 4 x 5 μL drops of spheroid suspension were transferred to a glass microscope slide and spheroids were counted at x4 magnification under an inverted light microscope (Olympus^®^ CK40-SLP). Spheroid suspensions were transferred to a sterile reagent reservoir, agitated with a multi-channel pipette to maintain a homogenous suspension and transferred to 96-well pHEMA-coated micro plates (Iwaki, Sterilin, UK) at a seeding density of 100 spheroids well^-1^ (in 75 μL L-15 medium; pH 7.4). Morphological integrity of spheroids was assessed and disaggregated or fused spheroids removed as a quality control check.

### Tetrazolium salt reduction (WST-1) viability assay

Spheroid viability was determined prior to substrate depletion experiments by a tetrazolium salt reduction method (WST-1 reagent, Roche Scientific, UK). Final well test concentrations of each pharmaceutical were 0, 32, 100 and 320 μg L^-1^; 0.1% DMSO; *n* = 6 wells per concentration). A solvent control (1% Triton X-100; 0.1% DMSO) was also included in the assay (*n* = 6 wells), as was a positive control (propranolol 100 μg L^-1^). Plates were incubated at 15°C for 48 h. After 24 h of exposure, 15 μL of WST-1 reagent was added directly to each well (1:10 dilution) and plates incubated for a further 24 h. Absorbance was read in a micro-plate reader (SpectraMax M5, Molecular Devices, USA) at 450 nm.

### Substrate depletion assay

Spheroids prepared from two separate livers were exposed in parallel on separate micro plates. To each row of a 96-well micro plate the following was added in 75 μL aliquots: (a) exposure medium (spheroids + media & pharmaceuticals; *n* = 6 wells); (b) solvent control medium (spheroids + media & solvent; *n* = 3 wells) and (c) exposure medium controls (pharmaceuticals—spheroids; *n* = 3 wells). Each micro plate row was allocated a time-point (0, 0.5, 1, 2, 4, 24 h; *n* = 12 wells) and substrate depletion quenched with the addition of 150 μL acetonitrile (ACN) at each respective time-point (in the instance where substrate depletion was not measured after 24 h, exposures were continued to ≤72 h). Plates were sealed to eliminate evaporative losses and analysed for substrate depletion by liquid chromatography with tandem mass spectrometry (LC-MS/MS). All assays were performed at a physiologically relevant temperature for rainbow trout (15°C ± 1°C). In summary, compounds were tested individually against spheroids generated from at least two individual fish livers, and chemical analysis conducted on samples from at least six time points.

### LC-MS/MS analysis

Standard solutions were prepared for each pharmaceutical to cover the range 1 to 1000 nM. These were made up to match the sample solvent composition i.e. 80:20 water:ACN containing 10 nM internal standard. Analyses were performed using a TSQ Quantum Access mass spectrometer (Thermo Scientific, San Jose CA, USA). Chromatographic separation was achieved by gradient elution on a Hypersil Gold 2.1 x 50 mm 3 μm C_18_ column (Thermo Scientific, San Jose CA, USA). The mobile phase was a mixture of (A) 0.1% formic acid in water and (B) 0.1% formic acid in methanol programmed as follows: 80% A to 100% B over 1.5 min and held for 1.5 min, then reset to initial conditions. The flow rate was 500 μL min^-1^ with an injection volume of 20 μL. The mass spectrometer was operated in electrospray ionization mode using selected ion monitoring with a capillary temperature of 270°C, vaporiser temperature 350°C, spray voltage 3750 V, sheath gas nitrogen @ 50 (arbitrary units) and auxiliary gas nitrogen at 30 (arbitrary units). Each compound was automatically optimized for ion polarity, precursor ion, and product ion and collision energy using QuickQuan software (Thermo Scientific San Jose CA USA).

### Data analysis

The depletion of parent substrate from the culture medium was determined by plotting measured concentration of test chemical (μg L^-1^) vs. incubation time (h^-1^). The depletion rate constant (*k*; h^-1^) was calculated by non-linear regression analysis (Sigma Plot 12.5, Systat Software, San Jose, USA), using a two parameter, exponential decay equation (y = ae^-bx^). The half-life (t_1/2_) for the exponential decay of the parent substrate was calculated using the depletion rate constant (t_1/2_ = (ln2)/k). Rate constants were divided by total cell number which approximated to 50,000 cells well^-1^—based on approx. 500 cells spheroid^-1^ and 100 spheroids well^-1^, (we have previously measured variability [10]) to calculate *in vitro* intrinsic clearance (Cl_INT, IN VITRO_ mL h^-1^ cell^-1^; adapted from [2, 14]). This data was then extrapolated to calculate intrinsic hepatic clearance (Cl_INT, HEPATIC_ mL h^-1^ g liver^-1^) to facilitate direct comparison with human data in the literature. For viability assays, datasets were analysed using one-way ANOVA with post-hoc Tukey HSD test (Minitab v15, Minitab Inc, USA).

## Results and Discussion

### Pharmaceutical metabolism

**Fig 1:**
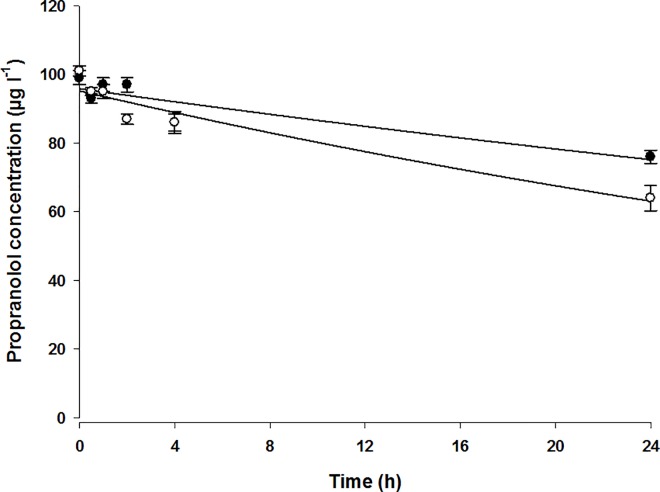
Substrate depletion kinetics of propranolol by trout liver spheroid cultures prepared from two separate fish livers. Closed circles denote cultures from fish one; open circles denote cultures from fish two (*n* = 6 at each time point). Values at each time point are mean ± SE. Substrate depletion kinetics determined using two-parameter, exponential decay curve-fit analysis (non-linear regression; Sigma Plot v12.5, Systat Software, San Jose, USA).

Pharmaceuticals representing four different drug families were chosen based on (a) their reported presence in the aquatic environment [6] and/or (b) biotransformation and bioaccumulation criteria taken from published human, mammalian and fish literature (see Tables 1, 2 & 3). A description of the pharmaceuticals and substrate depletion data is shown in Table 1. Substrate depletion kinetics and depletion rate constants for each pharmaceutical were determined using exponential decay curve-fit analysis (non-linear regression; Fig 1). Substrate depletion kinetics in spheroid cultures do not appear to be strictly linear, therefore we utilised a non-linear regression to calculate depletion constants. Propranolol metabolism was measured in multiple substrate depletion experiments (% loss: 41 ± 15.8; *n* = 12 fish) (Table 4). In comparison, the β-blockers atenolol (*n* = 4 fish) and metoprolol (*n* = 4 fish) demonstrated no evidence of substrate depletion in the 24 h exposure period, which was further examined at extended time points ≤48 h (atenolol) and ≤72 h (metoprolol). Propranolol was metabolised by spheroids from these individual fish run concurrently as a positive control for all compounds. Besides the current study and two others [2, 20], little is currently published on the metabolism of β-blockers in fish, therefore, we have little choice but to rely on extrapolated metabolism / pharmacokinetic data from mammalian studies to aid in our prediction of biotransformation and clearance of pharmaceuticals in fish [4]. Although their chemical structures share a number of similarities, the variation around the aromatic ring leads to a number of pharmacokinetic differences between different β-blockers. These include rate of uptake, lipid solubility, degree and rate of first-pass metabolism in the liver, binding to plasma proteins, half-life, and renal clearance of the drug and/or its metabolites [21, 22].

**Table 1:** Pharmaceuticals used in substrate depletion experiments using trout liver spheroids. Substrate decrease over total incubation period (%), depletion rates constant (*k*; h^-1^) and half-life (t_1/2_) values are shown as mean ± SD. NSD = no substrate depletion.

Pharmaceutical	Category	Log *K*_ow_ (pH)	% decrease over total	Depletion rate	Half-life hours (t_1/2_)
			incubation period	constant (*k*; h^-1^)	
Atenolol	Selective	0.02 (7.4)	NSD	-	-
	beta blocker				
Carbamazepine	Anticonvulsant	2.47 (7.0)	NSD	-	-
Diazepam	Benzodiazepine	2.70 (7.0)	NSD	-	-
	anxiolytic				
Diclofenac sodium	NSAID	4.02 (7.0)	39.3 ± 12.0	0.021 ± 0.008	39.2 ± 22.4
salt					
Metoprolol	Selective	0.09 (7.4)	NSD	-	-
succinate	beta blocker				
Phenylbutazone	NSAID	3.16 (7.4)	34.4 ± 12.9	0.015 ± 0.006	51.0 ± 22.9
Propranolol HCl	Non-selective	0.72 (7.0)	41 ± 15.8	0.022 ± 0.010	39.4 ± 23.9
	beta blocker				

**Table 2:** Prediction of pharmaceutical metabolism in trout liver spheroids based on ‘read-across’ from human metabolism data. ^**†**^ Pharmaceuticals are ranked according to the Biopharmaceutics Drug Disposition Classification System (BDDCS) [23] where 1 = High solubility / extensive metabolism; 2 = Low solubility / extensive metabolism; 3 = High solubility / poor metabolism. ^**+**^ Major CYP enzymes believed responsible for the metabolim of pharmaceuticals in humans (modified from [2] with additional data sourced from [44–47].

Parent	Classification ^†^	% metabolised	Major CYP enzyme	Metabolism		Predicted
		**in humans** ^**†**^	in humans ^+^	Humans	Trout (spheroids)	
Atenolol	3	6.00	2D6	**X**	**X**	✔
Carbamazepine	2	99.50	3A4	✔	**X**	**X**
Diazepam	1	99.50	2C19 / 3A4	✔	**X**	**X**
Diclofenac sodium salt	1	99.50	Believed to be principly 2C and likely 2C9. Several minor enzymes such as 2C8, 2C18, 2C19, 2B6 producing a wide range of metabolites	✔	✔	✔
Metoprolol succinate	1	99.00	2D6	✔	**X**	**X**
Phenylbutazone	1	99.00	Likely 2D6, 2C19, 3A4	✔	✔	✔
Propranolol HCl	1	99.75	1A2 / 2D6	✔	✔	✔

**Table 3:** Comparison of intrinsic hepatic clearance rates (CL_INT, HEPATIC_) of propranolol and diclofenac by trout liver spheroids with trout, human S9 and human hepatocytes. Clearance rates for human S9 and hepatocytes are shown as mean ± SD and taken from studies reviewed previously [2]. Clearance rates for trout S9 are taken from two previous fish *in vitro* studies [2,20]. Where no SD is provided, the data are collated from multiple studies and the figures provided for an indication of comparable rates.

Pharmaceutical	CL_INT, HEPATIC_ (intrinsic hepatic clearance: mL h^-1^ g liver^-1^)			
	Trout S9	Trout spheroid	Human S9	Human hepatocyte
Propranolol HCl	78.5 ± 82.7	54.0	52.5 ± 19.1	82.2 ± 39.9
Diclofenac sodium	9.5	49.8	368.0 ± 201.4	256.8 ± 123.1
salt				

**Table 4:** Propranolol depletion over time (%) measured over 24h incubation, calculated depletion rate constants (*k*; h^-1^) and half-life (hours) (t_1/2_) for liver spheroid cultures from individual fish experiments. Values for each individual fish experiment are mean ± sd from combined spheroid cultures (*n* = 6 wells). Initial measured dose at time zero was 98 ± 4 μg/L (n = 72 wells). Individual differences between fish were analysed by the natural log transform of the % depletion (normally distributed) and a one-way anova with Tukey *post hoc* to identify individual fish (fish sharing the same letter A through D are not different to one another). Fish number 12 had significantly slower clearance than any other fish (p<0.001), but has not been excluded from the dataset.

Individual fish experiment	Propranolol depletion over 24h incubation time (mean % ± sd)	Depletion rate constant (*k*; h^-1^)	Half-life (hours) (t_1/2_)	Individual fish statistical differences
1	24 ± 5	0.010	70.0	D
2	36 ± 9	0.017	40.3	C,D
3	39 ± 10	0.020	34.1	B,C
4	68 ± 11	0.045	15.5	A
5	39 ± 5	0.019	36.5	B,C
6	51 ± 12	0.027	25.7	A,B,C
7	34 ± 4	0.018	39.6	C,D
8	56 ± 5	0.032	21.6	A,B
9	34 ± 4	0.017	41.0	C,D
10	52 ± 6	0.029	23.7	A,B,C
11	50 ± 7	0.029	24.0	A,B,C
12	15 ± 5	0.007	100.4	E
**Mean ± SD**	**41 ± 15.8**	**0.022 ± 0.010**	**39.4 ± 23.8**	

In mammals, relatively more hydrophobic β-blockers such as propranolol and metoprolol undergo extensive Phase I hepatic metabolism with ~90% of the parent excreted by the kidneys as metabolites in the urine [23, 24]. Hydrophilic atenolol does not undergo such extensive metabolism and is excreted predominantly as the parent compound [25] with typically 94% excreted unchanged in human urine [24]. It is therefore of little surprise that the spheroids appear not to metabolise atenolol in this study. In humans, similar to propranolol, metoprolol is metabolised and eliminated by several oxidation pathways, the major of which via O-demethylation and further oxidation to a carboxylic acid metabolite that in man accounts for ~65% of the dose [26]. Metabolism of propranolol is affected by genetic polymorphism for both CYP1A (mephenytoin hydroxylation) and CYP2D6 (debrisoquine hydroxylation) isozymes in the liver [27, 28]. Metoprolol metabolism in particular is significantly affected by debrisoquine hydroxylation polymorphism [27]. Previous genomic studies with both rainbow trout [29] and zebrafish [30] has demonstrated an absence of CYP2D6, which could explain the lack of metoprolol metabolism demonstrated here; however the metabolism of propranolol and diclofenac might suggest the presence of another enzyme with similar function (Table 2).

Variability of substrate metabolism between individual fish (Table 2) is inevitably as a result of inherent metabolic differences, particularly in terms of their physiology and genetic makeup, which could be driving different pathways to the metabolism of propranolol i.e. modulation between the three major pathways [31]. Potential differences in the viability and morphological integrity of spheroids could contribute to this variation in metabolism; however, morphological integrity was rigorously assessed prior to exposure as a quality assurance check. There was no effect of any of the pharmaceuticals on spheroid viability (≤ 320 μg L^-1^), measured using a tetrazolium salt reduction assay, in any of the repeated exposures. Our choice of single fish replicates in this experiment was to assess the importance of the differences between individual animals and possible differences within each culture batch over time that might potentially affect the rate of xenobiotic metabolism [18, 31]. We suggest this is likely to reflect differences among fish *in vivo* and could contribute to the five-fold measured variability in circulating propranolol concentrations already reported in trout [32]. Given our understanding of the variability between individuals for propranolol, and balancing this against the numbers of fish required, we suggest two fish (biological replicates) are likely to be appropriate for investigative studies.

The degree of substrate depletion was similar in both non-steroidal anti-inflammatory drugs (NSAID) diclofenac (% loss: 39.3 ± 12.0; *n* = 4 fish) and phenylbutazone (% loss: 34.4 ± 12.9 *n* = 3 fish). Metabolism of diclofenac has been demonstrated previously *in vitro* with rainbow trout liver microsomal fractions (S9) [2]. Both larval zebrafish (*Danio rerio*) [33] and juvenile rainbow trout [34, 35] have demonstrated diclofenac metabolism *in vivo* with measurable levels of phase I and II metabolites. Metabolites and un-metabolised diclofenac have also been detected in the bile of adult rainbow trout [36], although large variations in both up-take and metabolism were observed between individual animals. Like propranolol, diclofenac is substrate for more than one human CYP, including CYP1A2 and 2D6 [2]. Phenylbutazone has demonstrated extensive metabolism in both humans [37] and horses [38] previously, but we are aware of no studies except the current on fish. Little seems to be known about exactly which cytochrome enzymes are involved in metabolism of phenylbutazone, but it seems likely that 2D6, 2C19, 3A4 are the primary enzymes in man; phenylbutazone is a well-documented strong inhibitor of human liver microsomal CYP2C9 activity [39].

No substrate depletion of the anticonvulsant carbamazepine, or the benzodiazepine anxiolytic diazepam was measured in any experiments. No measurable metabolism of carbamazepine in fish liver S9 fractions, a substrate for CYP3A4 in humans, has also been reported recently [2]. These authors also suggest that differences in CYP3A specificity between trout and mammals could lead to an absence of CYP3A4-like activity in trout, and that trout and other fish species may metabolise some, but not all, mammalian CYP3A substrates (but see phenylbutazone above), instead utilizing enzymes from other CYP families (e.g. CYP1A). CYP3A4 has not been identified in rainbow trout previously [29]. Several studies have demonstrated the involvement of CYP2C and CYP3A sub-families in human liver microsomal diazepam metabolism, with 3-hydroxylation catalyzed primarily by 3A P450s and *N*-demethylation partially mediated by 2C P450s [40–42].

The approach of studying the metabolism of pharmaceuticals in aquatic organisms via the “read-across” of mammalian toxicity and detoxification systems is important when such little data exists on these systems in fish [3, 4, 21, 31, 43, 44]. With reference to published pharmaceutical metabolism data from humans and fish (both *in vivo* and *in vitro*) and with particular reference to the substrate / CYP relationship (Table 2), data acquired from this and from previous *in vitro* fish metabolism studies, highlights the degree of caution that must be taken when presuming a homology of metabolic pathways between aquatic and terrestrial species [2]. Given the species differences and physiological / environmental factors such as temperature, direct comparisons of biotransformation and kinetics remain to be further investigated. Of the seven pharmaceuticals tested, four (propranolol, diclofenac, phenylbutazone and atenolol) demonstrated predicted substrate depletion based on read-across to human metabolism data [2, 45–48].

The enzyme CYP1A2 acts on both propranolol and diclofenac as its major and minor substrate respectively, with the latter having several CYP enzymes implicated in its metabolism [CYP2B6, CYP2C8, CYP2C9, CYP2C19, CYP2D6 and CYP3A4] in humans [2]. Trout liver spheroids exhibited a lack of activity towards the major CYP2C9 substrate (metoprolol), as well as major CYP2C19 and CYP3A4 substrates (diazepam and carbamazepine respectively), suggesting that fish may lack these isoforms (particularly *in vitro*) as demonstrated previously in rainbow trout [29] and zebrafish [30]. Further, *in vitro* systems may require pre-incubation with known CYP-inducers in order to up-regulate biotransformation systems in order to quantify metabolism in these substrates. For instance, incubation of fluoxetine with fish liver microsomal fractions obtained from several fish species, including rainbow trout, demonstrates slow, variable and often undetectable metabolism [2, 49], unless fish are pre-exposed to the major CYP3A4 substrate carbamazepine [49]. Therefore, we propose that propranolol may be another suitable candidate CYP-inducer in co-exposure studies where CYP 1A and 2D pathways might be predicted, and co-exposure a possibly more ecologically relevant approach. Identification of CYP enzymes and / or similar isoforms warrants further investigation to determine the specific metabolic pathways responsible for pharmaceutical biotransformation in fish. As the rainbow trout genome has now been sequenced [50], identification of these CYP enzymes and isoforms may now be possible.

In line with established and current extrapolation protocols for *in vitro* fish metabolism models [2, 12–14, 19, 51–54], we calculated clearance rates for propranolol and diclofenac and compared them with human clearance rates derived from the literature (Table 3; modified from trout and human intrinsic hepatic clearance literature review [2]). The extrapolation factors of 120 x 10^6^ hepatocytes g liver^-1^ and 50 mg microsomal protein g liver^-1^ used previously to convert literature values from human studies to mL h^-1^ g liver^-1^ [55, 56], for comparison to calculated trout clearance rates [2] were adopted in this study. The extrapolation factor for hepatocyte number was normalised against that obtained from spheroid experiments (91 x 10^6^ hepatocytes g liver^-1^). A high degree of variability was observed between rates collected from trout S9, human S9 and human hepatocyte studies for both drugs [55, 56]. Further, the other studies on diclofenac metabolism in fish focus on metabolite identification in the bile, making direct comparisons impossible [35, 36]. However, based on the limited dataset available, and taking study variability into account, estimated intrinsic hepatic clearance for propranolol in trout liver spheroids are similar to values recorded for humans previously (Table 3).

The rate for diclofenac was lower, however, a high degree of variability was again observed in these human studies. Clearance rate for propranolol was ~5 fold higher in trout spheroids but similar for diclofenac when compared to trout S9 data, although these comparable datasets are limited. It is unclear at this point whether clearance rates calculated using S9 fractions are an underestimation when compared to whole tissue. Due to scarcity of comparable data, it is currently unclear if differences between the various methods and compounds are biologically meaningful or reproducible. A previous study [2] conducted an intra-laboratory comparison of fish microsome metabolism and saw both re-assuring similarity but also identified variation. Here we demonstrate biological variability between individuals and replication reproducibility for a model we would expect to be more like an *in vivo* situation

It is also difficult to know exactly the effects of isolating cells and short-term measures compared to long-term 3-D culture techniques on rate values. For trout liver, [16–17], direct comparisons of freshly excised tissue; isolated cells in suspension and spheroids in extended culture demonstrated, with some exceptions, that gene expression and transporters in 3-D structures approximate well to the excised tissue. This suggests that the model represents at least some of the aspects of the *in vivo* situation. Further studies at both *in vitro* and *in vivo* level are urgently required to determine the sensitivity of S9 and tissue models in predicting whole animal responses with respect to biotransformation. As yet we are in no position to begin to understand the scale of an appropriate conversion factor for translating *in vitro* to *in vivo* as is common practice in mammalian pharmacology.

This study highlights the importance in understanding the metabolism of target pharmaceuticals in the mammal to better understand the pharmacokinetics in fish. In addition to the data presented here, propranolol demonstrates transport across gill epithelium [22]; uptake into blood plasma [32]; comparable hepatic biotransformation enzyme activity (EROD) induction rates under both *in vitro* and *in vivo* exposure conditions [31]; measurable substrate depletion in *in vitro* systems [2, 20] and the present study; and low toxicity *in vivo* [32] in fish, and therefore would be suitable as a positive control chemical to investigate metabolism. As a consequence of our experience in these studies, a concentration of 100 μg L^-1^ and an exposure period of 24 h are proposed as suitable parameters for subsequent pharmaceutical exposures utilising trout liver spheroids. Traditionally, *in vitro* toxicology experiments often use 24 h time-points and we utilised this design to demonstrate for the first time the sustained metabolic clearance in 3-D trout spheroids. However from our data, although we demonstrate a consistent depletion over 24 h, an acute exposure period (4 h) may be sufficient to measure significant metabolism of propranolol. Further, we believe the spheroids generated are of appropriate size to maintain normoxic conditions [57]. The compromise is that concentrations must be both pharmacologically relevant and analytically quantifiable in small working volumes. Concentrations at environmentally relevant levels are rarely quantifiable inside fish and especially at low volume [4]. The time course of 24 h provides time for acclimation and the potential for both phase I and II metabolism, giving relevant data for future physiologically based pharmacokinetic (PBPK) modelling applications.

In addition, with such limited data available on *in vivo* pharmaceutical metabolism in fish, we stress the importance, but also caution, of utilising read-across and extrapolation data from human / mammalian studies to aid predictions of biotransformation and clearance in trout. Further extrapolation of *in vitro* data for BCF calculations; an understanding of CYP enzymes responsible for drug metabolism, and information on biotransformation in other target organs (particularly those on the route of exposure such as gill or intestine), is an essential research requirement to enable a more thorough *in vitro* assessment of the bioaccumulation potential of pharmaceuticals in fish. Equally, utilization of such laboratory data (both *in vivo* and *in vitro*) for comparison with bioaccumulation endpoints measured in field experiments [58], is an essential direction for chemical bioaccumulation research in aquatic organisms.

## Supporting Information

S1 Data Supplemental data containing pharmaceutical concentrations. (XLSX)
